# Printability of External and Internal Structures Based on Digital Light Processing 3D Printing Technique

**DOI:** 10.3390/pharmaceutics12030207

**Published:** 2020-02-28

**Authors:** Yan Yang, Yanjun Zhou, Xiao Lin, Qingliang Yang, Gengshen Yang

**Affiliations:** College of Pharmaceutical Science, Zhejiang University of Technology, Hangzhou 310058, China; yangyan10@zjut.edu.cn (Y.Y.); zhouyanjun0521@163.com (Y.Z.); 17816875532@163.com (X.L.); qyang@zjut.edu.cn (Q.Y.)

**Keywords:** digital light processing, external and internal structures, printability, implants, physiological channels

## Abstract

The high printing efficiency and easy availability of desktop digital light processing (DLP) printers have made DLP 3D printing a promising technique with increasingly broad application prospects, particularly in personalized medicine. The objective of this study was to fabricate and evaluate medical samples with external and internal structures using the DLP technique. The influence of different additives and printing parameters on the printability and functionality of this technique was thoroughly evaluated. It was observed that the printability and mechanical properties of external structures were affected by the poly(ethylene glycol) diacrylate (PEGDA) concentration, plasticizers, layer height, and exposure time. The optimal printing solutions for 3D external and internal structures were 100% PEGDA and 75% PEGDA with 0.25 mg/mL tartrazine, respectively. And the optimal layer height for 3D external and internal structures were 0.02 mm and 0.05 mm, respectively. The optimal sample with external structures had an adequate drug-loading ability, acceptable sustained-release characteristics, and satisfactory biomechanical properties. In contrast, the printability of internal structures was affected by the photoabsorber, PEGDA concentration, layer height, and exposure time. The optimal samples with internal structures had good morphology, integrity and perfusion behavior. The present study showed that the DLP printing technique was capable of fabricating implants for drug delivery and physiological channels for in vivo evaluation.

## 1. Introduction

Digital light processing (DLP) is a 3D printing technique, in which photopolymer monomers are crosslinked layer by layer. Unlike the stereolithography apparatus technique, which is based on a point laser, the DLP technique uses a digital projector as a light source, leading to significantly reduced printing time [1]. To date, DLP printing has been successfully used in biomedical fields, such as dental prothesis and tissue engineering [2], revealing its feasibility for developing a digital workflow for personalized medicine.

It is noteworthy that the DLP technique is capable of fabricating samples with predetermined specific external structures for personalized implants. For example, it could be used to generate dental models with high accuracy [3], and cartilage with proper and satisfactory biomechanical properties [4]. In addition, the DLP technique could also produce samples with predetermined internal structures for highly complex organs, including the trachea, heart, lung, and vasculatures [5]. Recently, the DLP technique has been used to prepare solid preparations for oral drug administrations. Paracetamol tablets with controllable drug-release behavior were printed following the adding of suitable pore-forming agents [1], and theophylline tablets with programed drug-release profiles were fabricated with varying surface areas [6]. However, samples with external and internal structures had different requirements on morphology and function (e.g., drug dissolution and biomechanical properties). Therefore, it is necessary to further study the printability of different structures based on DLP printing.

Generally, the essential factor affecting the printability of DLP is the photopolymerizable system, which is usually composed of photopolymer monomer, photoinitiator and other additives (i.e., photoabsorbers, pore-forming agents and plasticizers). To date, commonly used biocompatible photopolymers, such as poly(ethylene glycol) diacrylate (PEGDA) [7], poly(ethylene glycol) dimethacrylate [8], gelatin methacrylate [9], and poly(propylene fumarate) [10] have been successfully used in DLP printing. Among them, hydrophilic PEGDA was attractive material owing to its tunable mechanical and biological properties [11]. Moreover, the concentration of PEGDA tremendously affected the fidelity, mechanical strength and release properties of the printed samples [6]. Furthermore, a photoinitiator determined the light wavelength of the photocuring reaction [12] and played an important role in printing efficiency [13]. In contrast, photoabsorbers were able to delay the photocuring reaction and improve the printability of internal channels [14]. In addition, pore-forming agents (e.g., PEG, NaCl and Mannitol) accelerated and adjusted drug release from the highly crosslinked structure [1], whereas plasticizers (e.g., PEG and glycerol) promoted the flexibility and biomechanical properties of the printed samples [15]. Considering that external and internal structures have different requirements on the ingredients of a photopolymerizable system, further studies on their printability are necessary.

In addition, the printing parameters also exert a great impact on the photocuring process and printing fidelity. For example, exposure time, voxel depth, and over-curing depth significantly affected the curing behavior of different photosensitive resins [16]. Although sufficient light intensity and exposure time were essential for photocuring, excessive exposure time might reduce printing resolution owing to the overgrowth caused by light scattering [17]. Reduced layer height led to printed channels with higher circularity and greater geometric fidelity to those of the original model [14]. The UV post-curing process also led to shape distortion and thus reduction of dimension accuracy [18]. Furthermore, the printing parameters greatly impact the mechanical and dissolution properties of the produced samples. For example, pattern ratio, orientation and waviness of models affected the mechanical behavior of the printed hydrogels [19], whereas the specific surface area of models modulated the drug-release behavior of printed tablets [20]. Considering the various morphological and functional requirements of implants and physiological channels, further studies on the effect of printing parameters and model design are important.

This study aimed to fabricate 3D models with predesigned external and internal structures by DLP using PEGDA as the photopolymer monomer. Furthermore, the effects of additives, printing parameters, and model design on the printability and functionality of external and internal structures were thoroughly evaluated.

## 2. Material and Methods

### 2.1. Material

PEGDA (400, No. 20180605) was provided by Ryoji Chemical Co. (Shanghai, China). Diphenyl (2,4,6-trimethylbenzoyl) phosphine oxide (DPPO, No. C1809028), tartrazine (No. F1926074) and PEG (300, No. L1707087) were purchased from Aladdin Biochemical Technology Co., Ltd. (Shanghai, China). Methylene blue (No. 20130321) was obtained from SSS Reagent Co., Ltd. (Shanghai, China). Coccine was obtained from Dyestuffs Research Institute Co., Ltd. (Shanghai, China). Diclofenac sodium (DS, No. 142436317488) was purchased from Sendi Biological Co., Ltd. (Shenzhen, China). Ibuprofen (No. KH23) was purchased from Dibo Biotechnology Co., Ltd. (Shanghai, China).

### 2.2. Printability Evaluation of External and Internal Structures

PEGDA and DPPO (1% of PEGDA amount) were mixed to prepare the photocuring solution. Water was added to prepare printing materials with PEGDA at concentrations of 25% to 100%. Methylene blue, coccine, and tartrazine were added as photoabsorbers to improve the printability of internal structures. The printing materials were added into the resin tank of a DLP printer (NOVA3D 101; NovRobotics, Inc. Shenzhen, China) equipped with a 405 nm laser generator. Models were sliced using the Creation Workshop software with a layer height of 0.02–0.05 mm. The exposure time for the bottom three layers was 10–90 s, and exposure time for the other layers was 2–12 s.

**Figure 1:**
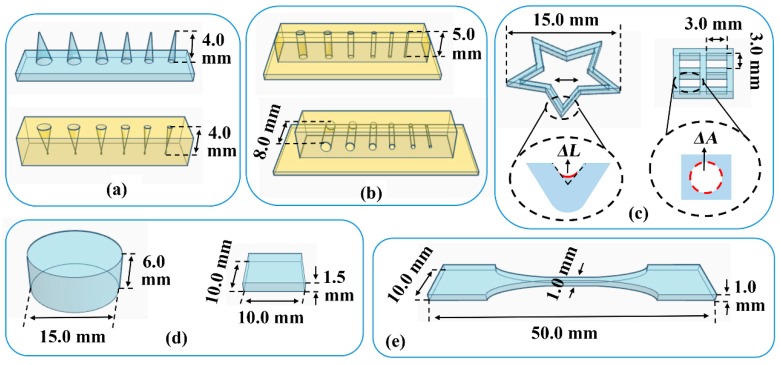
Models for printability evaluation and tensile test: (**a**) convex and concave cones; (**b**) vertical and horizontal channels; (**c**) pentagram and grid; (**d**) cylinder and cuboid; and (**e**) dumbbell-shaped. *ΔL*: inner arc length; *ΔA*: cavity area.

As shown in Figure 1a, convex and concave cones with different diameters (Φ = 2.0, 1.75, 1.5, 1.25, 1.0, and 0.75 mm) were printed to assess the printing accuracy of 3D external structures. The height of the convex cone or the depth of the concave cone was measured using a micrometer. The red solution was added to distinguish the boundary of the concave cones. As shown in Figure 1b, vertical and horizontal channels with different diameters (Φ = 1.75, 1.5, 1.25, 1.0, 0.75, and 0.5 mm) were printed to assess the printing accuracy of 3D internal structures. The red solution was added to distinguish the boundary of the channels, and the length of channels was measured using a micrometer. The printed length to design length ratio was defined as patency degree, and the minimum diameter of the channel with a patency degree of 100% was recorded as the minimum printable diameter (*D_min_*).

As illustrated in Figure 1c, pentagram and grid were printed to assess the printing accuracy of 2D structures. As an indicator of fusion at the inner corner, the inner arc length (*ΔL*) of the printed pentagram was measured using a stereomicroscope (ZY-HD1400; ZongyanWeiye, Shenzhen, China) with an S-EYE v1.1 measuring software. As an indicator of fusion at the edge, the cavity area (*ΔA*) of the printed grid was measured using the Image J software, and the cavity fusion rate (*R_f_*) was calculated. As illustrated in Figure 1d, the cylinder was printed to assess the substrate adhesion, and the printed height to the designed height ratio of the cylinder was calculated as the printing ratio. Whereas cuboid was printed to assess the photocuring degree and intensity, and the radial strength of the cuboid was determined using a hardness tester of tablets.

### 2.3. Mechanical Properties of the Printed Material

With the addition of PEG300 as a plasticizer, dumbbell-shaped samples (Figure 1e) were printed at a layer height of 0.05 mm and the exposure time of 60 s for bottom three layers and 8 s for other layers. The mechanical properties of the printed samples were evaluated using a previously described tensile test method [21] at a constant variable force of 0.008 N/s. The stress-strain curve was drawn and used in compression simulation [22]. The tensile stress at break (*σ_b_*) and elongation at break (*ε_b_*) were recorded to assess the strength and ductility of the material.

### 2.4. Preparation and Compression Behavior of Implants

**Figure 2:**
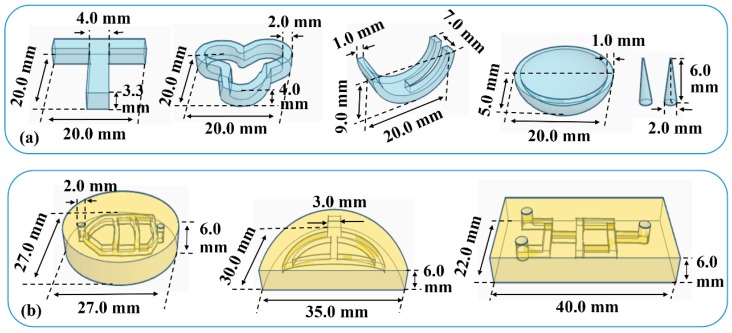
Models of (**a**) various implants and (**b**) physiological channels.

Various implants in Figure 2a were designed to verify the feasibility of printing samples with different external structures. Samples were printed at a layer height of 0.05 mm and the exposure time of 60 s for bottom three layers and 8 s for other layers. A constant compression force of 10 g was loaded on the surface of the printed implants to mimic the biomechanical behavior. The appearance of the implants before and after compression was compared.

Finite element method simulation was conducted to analyze the stress and strain behaviors of the implants under compression. Geometry models were established and meshed with PLAN82 elements using the ANSYS 10.0 software. The stress-strain curve of the printed material determined in Section 2.3 was inputted as nonlinear material properties, and the compression force was loaded on the surface of the implants. Von Mises stress (*σ_v_*) and Von Mises strain (*ε_v_*) distributions were simulated, and the overall deformation of the implants was analyzed.

### 2.5. In Vitro Dissolution of Implants

DS or ibuprofen (10%, *w*/*w*) was added in the printing formulation, and the drug-loaded implants in Figure 2a were printed at a layer height of 0.05 mm and the exposure time of 60 s for bottom three layers and 8 s for other layers. The specific surface area of implants was calculated by measuring the surface and volume. To compare the drug release behavior of implants with different shapes and applications, a simplified in vitro dissolution method was conducted at 37 ± 0.5 °C. According to literature, 10 mL deionized water [23] was used as release medium for implants loaded soluble drug DS, whereas 10 mL [24] phosphate buffer (pH = 7.2) [25] was used for implants loaded slightly soluble drug ibuprofen. At the predetermined time intervals, the dissolution medium was withdrawn and replaced with a fresh medium. The withdrawn sample was filtered through 0.45-μm Millipore filters and analyzed using a spectrophotometer (UV-2450; Shimadzu, Japan) at 274 nm for DS or 263 nm for ibuprofen. Cumulative drug release was calculated based on the validated DS calibration curve (A = 0.0325C − 0.0069, *R*² = 0.9993) or the validated ibuprofen calibration curve (A = 0.0018C + 0.0027, *R*² = 0.9998).

### 2.6. Preparation and Evaluation of Physiological Channels

Physiological channels in Figure 2b were designed to verify the feasibility of printing samples with different internal structures. Samples were printed at a layer height of 0.05 mm and the exposure time of 60 s for bottom three layers and 8 s for other layers. For the perfusion test, red and green solutions were added into the channel. Fluid flow behavior during the perfusion process was observed to evaluate the morphology and integrity of the internal channels.

Computational fluid dynamics (CFD) simulation was carried to analyze the flow of fluids and the interactions between liquids and channel surfaces. The 3D geometry model used was shown in Figure 2b, and the 2D cross-section of the channel inside the model (flow field) was established using the Gambit 2.4 software. The mesh model was established using Tri&Pave elements with a spacing of 20. The boundary conditions were defined as follows: the left borders were velocity-inlet, the right border was outflow, and the other borders were wall. The water density and viscosity at 25 °C were inputted as material properties, whereas the blood flow velocity of adults (0.14 m/s) was inputted as inlet velocity. Steady simulation of the flow fields [26] was conducted using the Fluent 6.3 software. A multiple reference frame model was used to simulate the axial flow, and a standard *κ-ε* model was adopted to describe the turbulence. The PRESTO! algorithm was used to calculate pressure and the SIMPLE algorithm was used to couple pressure and velocity.

## 3. Results and Discussion

### 3.1. The Effect of Photoabsorbers

**Figure 3:**
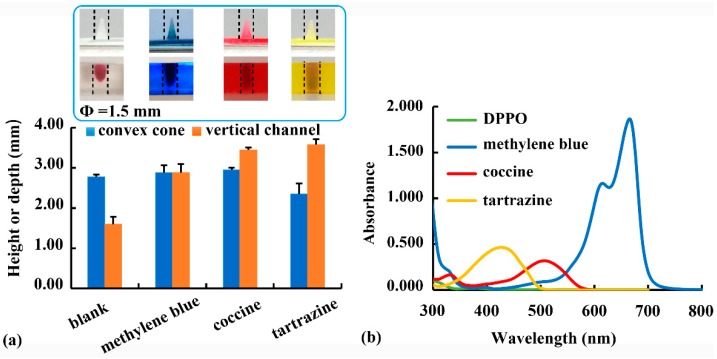
(**a**) Effect of different photoabsorbers (i.e., methylene blue, coccine, and tartrazine) on the printability of convex cone and vertical channel; (**b**) wavelength scan results of the photoinitiator (i.e., Diphenyl (2,4,6-trimethylbenzoyl) phosphine oxide, DPPO) and photoabsorbers.

Using PEGDA as monomer and DPPO (1%, *w*/*v*) as photoinitiator, the effect of different photoabsorbers (0.25 mg/mL, *w*/*v*) on the printability of external and internal structures was evaluated. As shown in Figure 3a, the height of the cone (Φ = 1.5 mm) was not different after the addition of methylene blue or coccine. However, the height of the cone decreased with the addition of tartrazine, revealing that tartrazine was able to slow down the light-induced crosslinking reaction [14]. Furthermore, the depth of the internal vertical channel (Φ = 1.5 mm) increased after different photoabsorbers was added, as photoabsorbers was able to minimize light scattering into the channel, thereby slowing down the over-curing of the non-printed area and facilitating the printability of internal structures.

The wavelength scanning results of photoinitiator and photoabsorber solutions (0.01 mg/mL, *w*/*v*) are shown in Figure 3b. At the printing wavelength (405 nm), the order of the absorbance values was as follows: tartrazine > coccine > methylene blue > DPPO. With the highest absorbance at 405 nm, tartrazine was the most effective photoabsorber in slowing down the photocuring reaction. Therefore, tartrazine addition had the most obvious reducing effect on cone printability but improving effect on channel printability (Figure 3a). Owing to its high photoabsorption efficiency and good biocompatibility, tartrazine should be added when printing internal structures. However, it was unnecessary for printing external structures. Besides, DPPO (1%, *w*/*v*) was found to be an effective photoinitiator of PEGDA polymers at the printing wavelength of 405 nm [15,27]. However, the determined absorbance of DPPO at 405 nm was much lower than those of photoabsorbers (Figure 3b). A similar phenomenon was found in the literature [14], and it might be related to the different effects of photoinitiator and photoabsorber on the photocuring process. Other photoinitiators with higher absorbance at 405 nm should be studied in further study.

**Figure 4:**
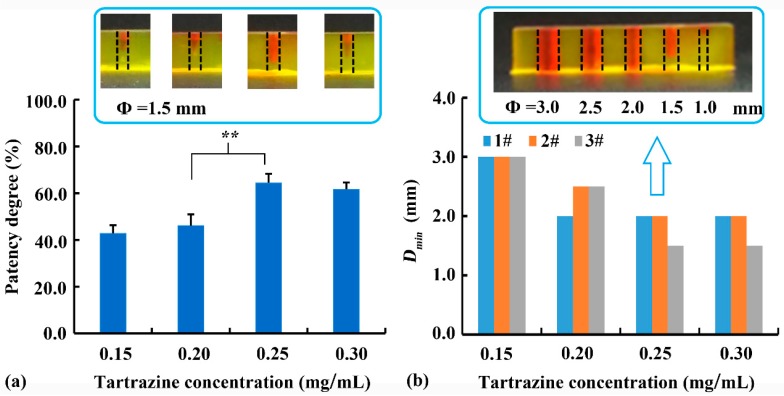
Effect of tartrazine concentration on (**a**) patency degree of the vertical channel with a diameter of 1.5 mm (** denote *p* < 0.01), and (**b**) the minimum printable diameter (*D_min_*) of the vertical channel with different diameters, and typical sample with 0.25% tartrazine was illustrated.

The effect of tartrazine concentration as a photoabsorber on the printability of the internal vertical channel was evaluated. As shown in Figure 4a, there was no significant difference (*p* > 0.05) in patency degree of 0.15 and 0.20 mg/mL tartrazine, or 0.25 and 0.30 mg/mL tartrazine. The reduction in average patency degree of 0.30 mg/mL tartrazine compared to that of 0.25 mg/mL tartrazine might due to the low printability (irregularly cylindrical internal channel) and high detection deviation. However, when tartrazine concentration increased from 0.20 mg/mL to 0.25 mg/mL, the patency degree significantly increased from 46.1% to 64.4% (*p* < 0.01), suggesting an improvement of printing accuracy. As shown in Figure 4b, a sample with different diameters and a height of 6 mm was printed to determinate the *D_min_*. Considering that the DLP printability is not linearly related to the *D_min_*, the *D_min_* of every printed sample (*n* = 3) were listed in Figure 4b instead of the average and standard deviation. When the tartrazine concentration increased from 0.15% to 0.25%, the *D_min_* of channel decreased. This improvement of printing accuracy of the internal channel was related to the absorption of light scattered into the channel by tartrazine. Addition of excessive tartrazine (0.3 mg/mL) could not further improve printability, suggesting the limited effect of a photoabsorber on printability. Therefore, the optimal tartrazine concentration of 0.25 mg/mL was used in further studies.

### 3.2. Effect of PEGDA Concentration

**Figure 5:**
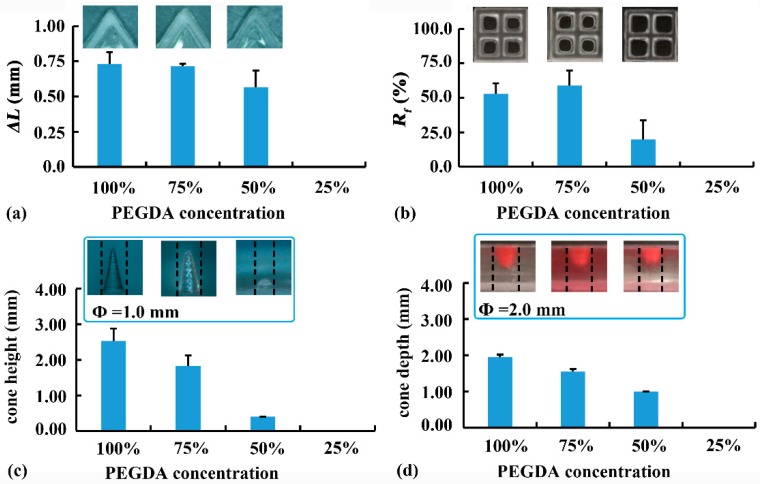
Effect of poly(ethylene glycol) diacrylate (PEGDA) concentration on the printability: (**a**) inner arc length (*ΔL*) and (**b**) cavity fusion rate (*R_f_*) of 2D samples; and (**c**) cone height and (**d**) cone depth of 3D samples.

The effect of PEGDA concentration on the printability of 2D samples is shown in Figure 5a,b. High PEGDA concentrations (i.e., 100% and 75%) led to large *ΔL* at the top layer of the pentagram (0.44 mm and 0.43 mm, respectively), and high *R_f_* at the bottom layer of the grid (52.9% and 58.9%, respectively). The undesirable crosslinking at the non-printed area was caused by the light scattered from the solidified edge. The exposure time of the bottom three layers (60 s) was much longer than that of the other layers (8 s). Therefore, more light was scattered at the bottom, leading to more serious over-curing at the bottom layers. With a low PEGDA concentration of 50%, both the *ΔL* of the pentagram and the *R_f_* of the grid decreased, revealing less fusion at the inner corner and edges. Further reduction of PEGDA concentration to 25% resulted in printing failure. Therefore, 50% PEGDA was preferred for 2D samples requiring high printing accuracy at the bottom.

The effect of PEGDA concentration on the printability of 3D samples is shown in Figure 5c,d. When the PEGDA concentration was reduced from 100% to 50%, the height of the convex cone (Φ = 1.0 mm) decreased from 2.53 mm to 0.40 mm, as low PEGDA concentration led to reduced crosslinking degree and negatively affected the printability of fine structure. In contrast, the concave cone (Φ = 1.0 mm) could not be printed using PEGDA solution (100–25%), suggesting that printing concave cone was more difficult than printing convex cone. When printing large concave cone (Φ = 2.0 mm), a similar effect of PEGDA concentration on printability was observed. Reduced PEGDA concentration decreased printability, and insufficient PEGDA concentration (25%) caused printing failure. Therefore, 100% PEGDA (Rx-I) was optimized for printing 3D samples with external structures.

**Figure 6:**
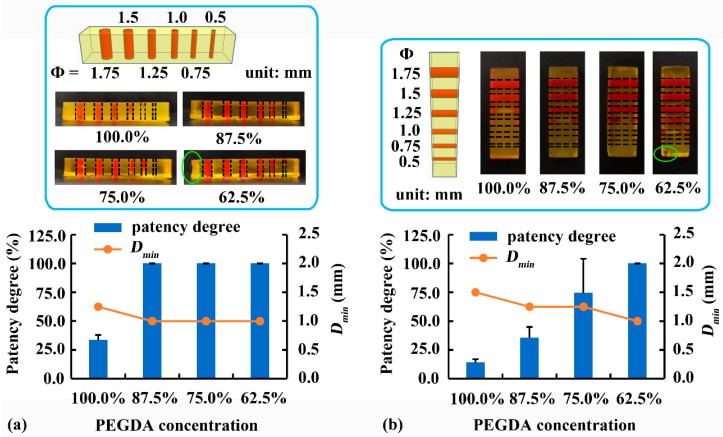
Effect of PEGDA concentration on the printability of (**a**) vertical and (**b**) horizontal channels. *D_min_*: minimum printable diameter.

To improve the low printability of internal structures in Figure 5d, 0.25 mg/mL tartrazine was added and the effect of PEGDA concentration on the printability was further studied. As illustrated in Figure 6a, reducing PEGDA concentration facilitated the printability of internal vertical channels (Φ = 1.75, 1.5, 1.25, 1.0, 0.75, and 0.5 mm). When PEGDA concentration decreased from 100% to 87.5%, the patency degree of the vertical channel (Φ = 1.0 mm) increased from 33.5% to 100%, and the *D_min_* of vertical channel decreased from 1.25 mm to 1.0 mm. Further reduction of PEGDA concentration to 75.0% or 62.5% had no significant effect on the printability of vertical channels. As illustrated in Figure 6b, reducing PEGDA concentration also improved the printability of internal horizontal channels (Φ = 1.75, 1.5, 1.25, 1.0, 0.75, and 0.5 mm). When PEGDA concentration decreased from 100% to 62.5%, the patency degree of the horizontal channels (Φ = 1.0 mm) increased from 14.1% to 100%, and the *D_min_* of horizontal channels decreased from 1.5 mm to 1.0 mm.

The horizontal channel was perpendicular to the projection direction of light [14]; therefore, a large amount of light penetrated through the transparent crosslinked area into the channel, causing over-curing inside the channel. In contrast, the vertical channel was parallel to the projection direction of light, leading to a small amount of light scattered into the channel. Therefore, reducing PEGDA concentration was more effective in minimizing over-curing inside horizontal channels than inside vertical channels. Although 62.5% PEGDA with 0.25 mg/mL tartrazine led to the highest printability of internal channels, edge deformation and corner defects were observed (green circle) in Figure 6a,b. Therefore, 75% PEGDA with 0.25 mg/mL tartrazine (Rx-II) was the optimal printing solution for samples with internal channels.

### 3.3. Effect of Layer Height

**Figure 7:**
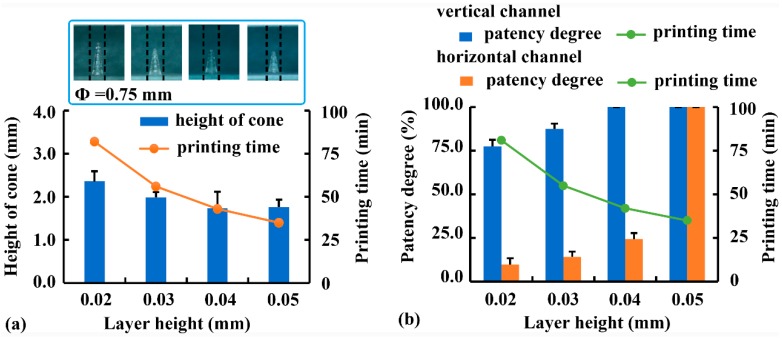
Effect of layer height on the printing accuracy and efficiency of (**a**) convex cones and (**b**) internal channels.

Using Rx-I as a printing solution, the effect of layer height on the printability of samples with external structures was investigated, and the results are illustrated in Figure 7a. As layer height increased from 0.02 mm to 0.05 mm, the height of convex cones (Φ = 0.75 mm) decreased from 2.36 mm to 1.76 mm, and the printing time was decreased from 82 min to 35 min. This was consistent with the results of FDM printing [28], in which reduced layer height was beneficial to printing accuracy but adverse to printing efficiency. Therefore, the optimal layer height for the external structures was determined to be 0.02 mm.

Using Rx-II as a printing solution, the effect of layer height on the printability of samples with internal structures was investigated (Figure 7b). As layer height increased from 0.02 mm to 0.05 mm, the patency degree of vertical channels (Φ = 1.0 mm) increased from 77.5% to 100.0%, whereas the printing time decreased from 81 min to 35 min. The improved printability of vertical channels with increased layer height was due to shorter exposure time and less amount of light scattered into the channel. Although the printability of horizontal channels was lower than that of vertical channels, increasing layer height was more effective in minimizing over-curing inside horizontal channels. When layer height increased from 0.02 mm to 0.05 mm, the patency degree of the horizontal channel (Φ = 1.0 mm) increased from 9.7% to 100.0%. Because the samples had the same height, the printing time of horizontal channels was not different from that of vertical channels. Considering both printing accuracy and efficiency, 0.05 mm was the optimal layer height for internal channels within the range of 0.02 mm and 0.05 mm.

### 3.4. Effect of Exposure Time

**Figure 8:**
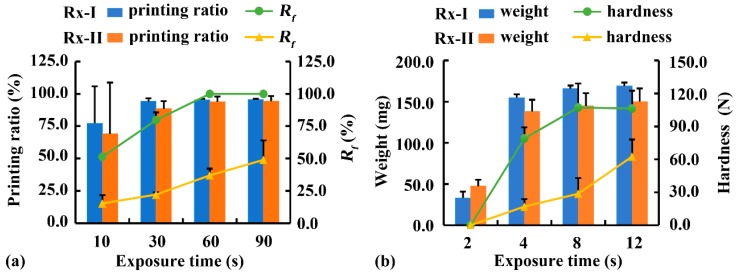
Effect of exposure time of (**a**) bottom three layers and (**b**) other layers on printability. Rx-I: 100% PEGDA; Rx-II: 75% PEGDA with 0.25 mg/mL tartrazine; *R_f_*: cavity fusion rate.

With a constant exposure time of other layers (8 s), the effect of exposure time of the bottom three layers on substrate adhesion was evaluated using the cylinder with a diameter of 15 mm and a height of 6 mm (Figure 1d). As shown in Figure 8a, when the cylinder was printed using Rx-I at an exposure time of 10 s, substrate adhesion was insufficient to complete the printing and the printed sample fell off the printing platform with a printing ratio of 77.3%. With prolonged bottom exposure time from 30 s to 90 s, substrate adhesion was sufficient for printing, although more force was required to demold the printed samples. A similar tendency was observed when a cylinder was printed using Rx-II as a printing solution. When the bottom exposure time was 10 s, the printing ratio of Rx-II (69.1%) was slightly lower than that of Rx-I (77.3%), indicating that the water and photoabsorber in Rx-II decelerated the crosslinking reaction and negatively affected on substrate adhesion. Cylinders with a constant height of 6 mm and different diameters (i.e., 5, 10, and 15 mm) were printed to study the effect of diameters on surface adhesion. The results (not provided) revealed that the different diameters had no significant influence on surface adhesion per interfacial area. However, it was difficult to evaluate the surface adhesion of the grid with a small height of 1.5 mm (Figure 1c). No grid sample fell off during printing at the exposure time of 10 s. It was related to the small height and the serious cavity fusion, which greatly increased the interfacial area.

The effect of bottom exposure time on the fusion of grid is illustrated in Figure 8a. The *R_f_* of the grid (2 × 2 mm^2^) increased from 54.1% to 100% when it was printed using Rx-I with prolonged exposure time from 10 s to 90 s. A similar tendency was observed when the grid was printed using Rx-II, although the *R_f_* of Rx-II was much lower than that of Rx-I, because of the adverse effect of the water and photoabsorber in Rx-II on over-curing. In short, the bottom exposure time was optimized to 60 s to guarantee sufficient substrate adhesion during printing. However, when a structure with porous structures at the bottom was printed, the bottom exposure time should be reduced.

In addition, the effect of exposure time of other layers on photocuring degree and intensity was studied at a constant bottom exposure time of 60 s (Figure 8b). When a cuboid structure (Figure 1d) was printed using Rx-I with increased exposure time from 2 s to 8 s, the weight increased from 33 mg to 166 mg and the hardness increased from 0 N to 107.2 N. Further prolongation of exposure time to 12 s led to no difference, suggesting that exposure to light for 8 s was sufficient to complete the photocuring reaction. A similar tendency was observed when a cuboid was printed using Rx-II, although the weight and hardness decreased, owing to the adverse effect of the water and photoabsorbter in Rx-II on photocuring. Considering the photocuring degree and printing efficiency, the optimal exposure time for other layers was 8 s.

### 3.5. Compression Behavior and Simulation of Implants

**Figure 9:**
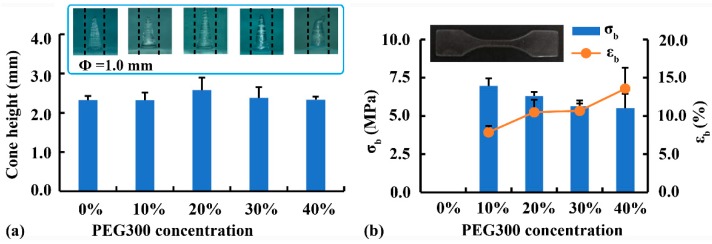
Effect of PEG300 concentration on (**a**) the printability of fine structures and (**b**) the mechanical properties of printed materials.

To mimic the biomechanical properties of implants, PEG300 was added as a plasticizer to improve the flexibility of crosslinked PEGDA [15]. The effect of PEG300 concentration on the printability of convex cone (Φ = 1.0 mm) is shown in Figure 9a. Cone height was the highest after the addition of 20% PEG300, whereas the cone deformation was the most serious after the addition of 40% PEG300. This finding suggested that a moderate amount of plasticizer was beneficial to printability, whereas an excessive amount of plasticizer caused poor strength and deformation. The effect of PEG300 concentration on the mechanical properties of printed materials is shown in Figure 9b. Printed samples without PEG300 were too brittle to be completely demold and evaluated by the tensile test. As PEG300 increased from 10% to 40%, *σ_b_* decreased from 7.0 MPa to 5.5 MPa, whereas *ε_b_* increased from 7.9% to 13.6%. The added PEG interspersed within the PEGDA chains, thus reducing the degree of crosslinking between PEGDA chains [15]. Considering the printing accuracy and mechanical properties, the optimal printing solution for implants was PEGDA:PEG300 = 80:20 (Rx-III).

**Figure 10:**
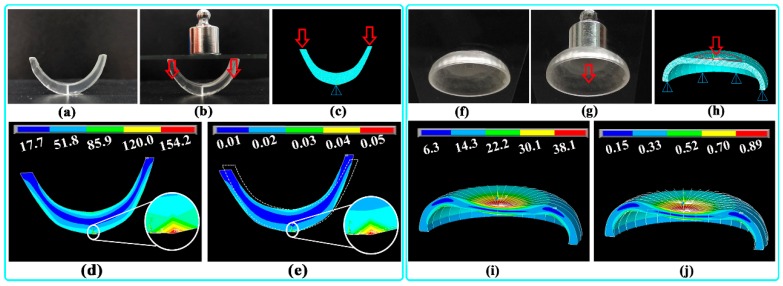
Typical U-shaped sample: (**a**) before and (**b**) after compression; (**c**) mesh model with loaded pressure; simulated (**d**) Von Mises stress (*σ_v_*) and (**e**) Von Mises strain (*ε_v_*) distribution. Typical arc-shaped sample: (**f**) before and (**g**) after compression; (**h**) mesh model with loaded pressure; simulated (**i**) *σ_v_* and (**j**) *ε_v_* distribution.

The optimal printing material (Rx-III) showed obvious plastic deformation during the tensile test (ε_b_ >10%); thus, the Von Mises criterion was used as a failure criterion during compression behavior simulation. As shown in Figure 10a,b, the printed U-shaped sample had good strength and elasticity under a compression force of 10 g, and the deformation was recovered after the compression force was removed. A typical 2D cross-section mesh model of the U-shaped sample is shown in Figure 10c; the vertical pressure loaded on the upper surface of the sample was 8.6 kPa. The simulated *σ_v_* distribution (Figure 10d) revealed that the bottom of the sample withstood the most stress (labeled as a red region). The ultimate *σ_v_* (154.2 kPa) was much lower than the *σ_b_* of the material (6.3 MPa) shown in Figure 9b; therefore, the integrity of the printed sample could be guaranteed. Furthermore, the simulated *ε_v_* distribution (Figure 10e) suggested that the bottom of the sample withstood the most strain (labeled as a red region), with the ultimate *ε_v_* of 0.05%. The deformed shape was also compared with the undeformed shape (marked with white dashed lines), and the overall deformation and displacement of the sample could be visualized.

As shown in Figure 10f,g, the printed arc-shaped sample had good strength and elasticity under a compression force of 10 g, and the deformation was recovered after the compression force was removed. A typical 3D mesh model of the arc-shaped sample is shown in Figure 10h; the vertical pressure loaded on the upper surface of the sample was 1.35 kPa. The simulated *σ_v_* distribution (Figure 10i) revealed that the upper surface of the sample withstood the most stress (labeled as a red region). The ultimate *σ_v_* (38.1 kPa) was much lower than the *σ_b_* of the material (6.3 MPa) shown in Figure 9b; therefore, the integrity of the printed samples could be guaranteed. Furthermore, the simulated *ε_v_* distribution (Figure 10j) suggested that the upper surface of the sample withstood the most strain (labeled as a red region), with the ultimate *ε_v_* of 0.89%. The deformed shape was also compared with the undeformed shape (marked with white border lines), and the overall deformation of the sample could be visualized.

### 3.6. In Vitro Dissolution of Implants

**Figure 11:**
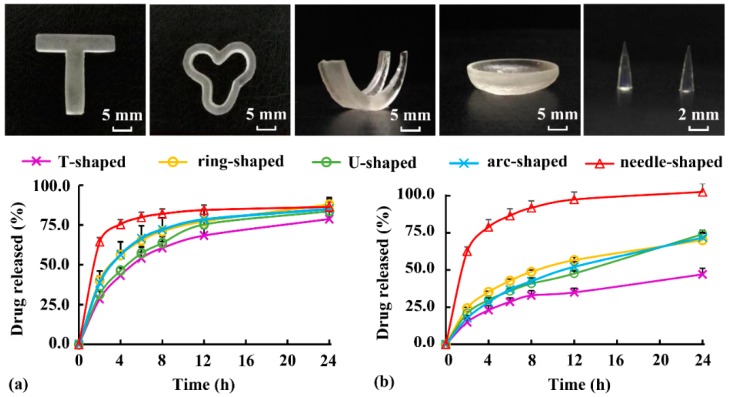
Appearance and dissolution behavior of implants loaded with (**a**) diclofenac sodium (DS) and (**b**) ibuprofen.

As typical soluble and insoluble drugs, respectively, DS and ibuprofen were added into Rx-III to evaluate the drug-loading ability and dissolution behavior. Various implants (Figure 2a) with a length of 20 mm and a weight of approximately 500 mg were printed. As shown in Figure 11, T-shaped and ring-shaped samples could be used as intrauterine devices, U-shaped and arc-shaped samples could be applied as femoral cartilage and contact lens, and the needle-shaped sample had application potential as a minimally invasive implant. According to the printability of external structures, the smallest diameter of cone printed by DLP was about 0.5 mm. Therefore, microneedle (Φ < 0.3 mm) could not be printed using the DLP method in this study.

As shown in Figure 11a, the DS-release behavior of various implants exhibited sustained-release characteristics. The DS released from the needle-shaped samples had the highest release speed and degree during 24 h, owing to its highest specific surface area (Table 1). It was agreed with the literature [20], where the drug release from printed tablets was dependent on the surface area to volume ratio. As shown in Figure 11b, ibuprofen release from the implants was much slower than DS release, and the sustained-release behavior might last for several days (except for ibuprofen release from the needle-shaped samples). The results showed that both soluble and insoluble drugs could be loaded into Rx-III, and the sustained-released behavior of the printed implants could be adjusted by changing the specific surface area. However, it was found that the highly soluble PEG300 added as plasticizer leached out and greatly accelerated the drug release behaviors in Figure 11. Therefore, PEG300 was not the best choice for biomedical implants requiring long-acting medication, and it was used in this study to evaluate the influence of different shapes on dissolution. To accurately assess the sustained-release behavior of implants, water-insoluble plasticizers should be further studied, and the in vitro dissolution method should be modified to better mimic the implant environment in the body.

### 3.7. Perfusion Behavior and Simulation of Physiological Channels

**Figure 12:**
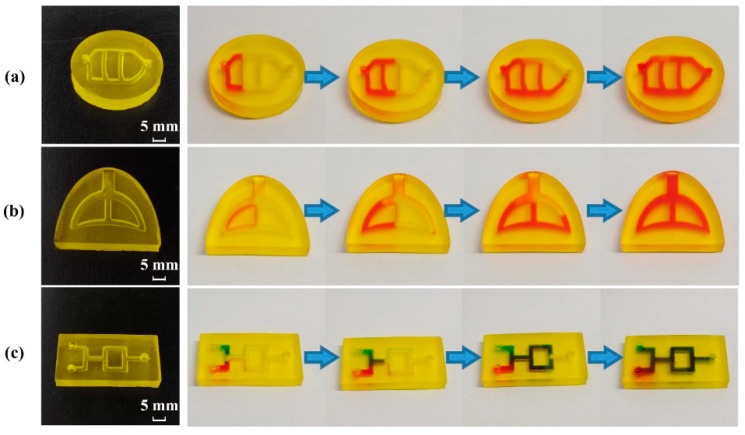
Printed samples with internal channels and their perfusion process: (**a**) vascular model, (**b**) tracheal model, and (**c**) fluid mixing model.

Typical samples (Figure 2b) were printed to mimic blood vessels and trachea using optimal printing material for internal channels (Rx-II). As shown in Figure 12a, the vascular model was successfully printed, and the perfusion process showed the good morphology and integrity of the internal channel. The vascular model could be used as an in vitro platform to evaluate the elution performance, diffusion behavior and biological perfusion. As shown in Figure 12b, a tracheal model with good morphology was printed, and the integrity of the sample was proved by a perfusion test. To mimic the trachea and bronchi, the inlet and outlet of simplified model overlapped at a large channel (Φ = 3 mm). However, the ductility and gas permeability of this model should be improved before it could be used as a simplified platform to evaluate the biomechanical properties of the trachea and the powder deposition process during pulmonary administration.

In addition, a fluid mixing model with good morphology and integrity is illustrated in Figure 12c. The green and red solutions were separately added into the inlets on the left. When the solutions flowed along the internal channels, the green and red solutions mixed, separated, remixed, and then flowed out from the outlet on the right. The mixing performance and hydrodynamic behavior of multiple fluids could be evaluated using this model. According to the printability of internal structures, the smallest diameter of channel printed by DLP was about 1.0 mm. Therefore, microfluidic chip (Φ < 0.1 mm) could not be printed using the DLP method in this study.

**Figure 13:**
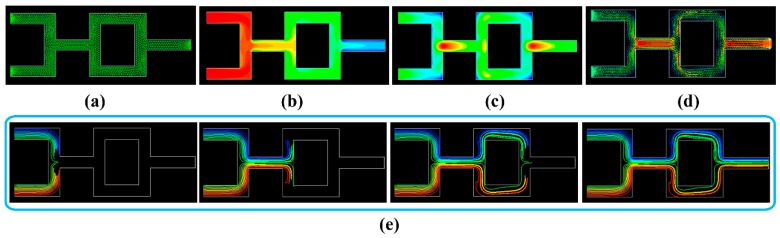
Simulated 2D cross-section flow field of the fluid mixing model: (**a**) mesh model; (**b**) total pressure contour; (**c**) turbulent intensity contour; (**d**) velocity vector; and (**e**) partial pathlines at different steps colored by particles.

The fluid mixing model in Figure 12c was used for CFD simulation, and its 2D cross-section flow field is shown in Figure 13a. To simplify the simulation, a multiple reference frame model was adopted to simulate the steady-state of the fluid field. As illustrated in Figure 13b, the simulated total pressure decreased along the fluid field from the entrance (212 Pa) to the exit (1.3 Pa). The simulated turbulent intensity (Figure 13c) was distributed in a non-uniform manner, and the maximum turbulent intensity appeared at the position (marked in red) where the two liquids intersected. The fluid velocity (Figure 13d) was also non-uniformly distributed, and the maximum velocity appeared at the position (marked in red) where these two liquids mixed together. Furthermore, the partial pathlines (Figure 13e) were simulated to visualize the two-liquid mixing process, and the results at different steps agreed with the results of the perfusion process in Figure 12c. The CFD simulation results could be used to further study the influence of material strength and channel design on the mixing performance.

## 4. Conclusion

Medical samples with specialized functional structures were successfully fabricated and thoroughly evaluated by utilizing the DLP technique. On one hand, under the optimal printing conditions for external structures, various implants were successfully produced, and their potentials as implants for drug delivery were proven. On the other hand, under the optimal printing conditions for internal structures, various physiological channels were successfully prepared, and their application in perfusion evaluation was confirmed. Further studies are still necessary to investigate the possibilities of improving the printing accuracy, and the in vivo evaluation methodology should be established to further illustrate the biomechanical behavior and flow field of the printed implants.

## Figures and Tables

**Table 1:** The specific surface area of various implants.

	T-Shaped	Ring-Shaped	U-Shaped	Arc-Shaped	Needle-Shaped
surface area (m^2^)	5.8 × 10^−4^	5.8 × 10^−4^	4.7 × 10^−4^	7.5 × 10^−4^	2.2 × 10^−5^
volume (m^3^)	4.2 × 10^−7^	4.2 × 10^−7^	4.6 × 10^−7^	5.8 × 10^−7^	3.6 × 10^−9^
specific surface area (m^2^/m^3^)	1.4 × 10^3^	1.4 × 10^3^	1.0 × 10^3^	1.3 × 10^3^	6.2 × 10^3^

## Nomenclature

DLP	digital light processing
PEGDA	polyethylene glycol diacrylate
DPPO	diphenyl(2,4,6-trimethylbenzoyl) phosphine oxide
DS	diclofenac sodium
*D_min_*	the minimum printable diameter
*ΔL*	inner arc length
*ΔA*	cavity area
*R_f_*	cavity fusion rate
*σ_b_*	tensile stress at break
*ε_b_*	elongation at break
*σ_v_*	Von Mises stress
*ε_v_*	Von Mises strain
CFD	computational fluid dynamics
Rx-I	100% PEGDA
Rx-II	75% PEGDA with 0.25 mg/mL tartrazine
Rx-III	PEGDA: PEG300 = 80: 20
